# Clinico-radiological outcome of Arthroscopic Anterior Cruciate Ligament Reconstruction with Augmentation of Dehydrated Human Amnion Chorion Allograft Membrane using Peroneus Longus Autograft

**DOI:** 10.5704/MOJ.2403.005

**Published:** 2024-03

**Authors:** PB Tonape, JVS Kishore, RM Kopparthi, T Tonape, DS Bhamare, S Desireddy

**Affiliations:** 1 Department of Orthopedics, Sterling Multispeciality Hospital, Pune, India; 2 Department of Orthopedics, Dr. D. Y. Patil Medical College, Hospital and Research Centre, Pune, India; 3 Department of Radiology, Dr. D. Y. Patil Medical College, Hospital and Research Centre, Pune, India; 4 Department of General Surgery, Dr. D. Y. Patil Medical College, Hospital and Research Centre, Pune, India; 5 Department of Research, Pune, India

**Keywords:** anterior cruciate ligament reconstruction, dehydrated human amnion chorion membrane, allograft, signal-to-noise ratio, tegnor lysholm score

## Abstract

**Introduction:**

For many sportsmen, anterior cruciate ligament (ACL) tears are unfortunate but common injuries. Several growth factors, cytokine, chemokine, and protease inhibitors functions in stimulation of paracrine reactions in fibroblast, endothelial, and stem cells thereby promoting the tissue restorative processes. Augmented with dehydrated Human Amnion Chorion Membrane (dHACM) allograft reinforces the reconstructed ligament and aids in effective restoration.

**Materials and methods:**

In this case control study 15 patients undertaking ACL reconstruction with tripled peroneus augmented dHACM (G1) were prospectively monitored up for a period of 8 months along with 15 control patients (G2) without dHACM augmentation. Clinical and radiological outcomes were analysed and assessed about effect of augmenting the peroneus longus graft using dHACM. Clinical analysis included pre-operative two, four, six, and eight months post-operative Tegnor-Lysholm score, and radiological analysis included the 6th month postoperative MRI signal-to-noise ratio (SNR) measurements by mean signal-value at femoral insertion, midsubstance and tibial insertion of ACL graft.

**Results:**

Clinically, as a mean Lysholm score of all patients, they were revealed to be consecutively high in G1 than in Group 2 at four, six, and eight months. The signal-to-noise ratio from the MRI results showed majority having good healing in G1 group.

**Conclusions:**

Based on 6-month MRI, an effective ligamentization (SNR<75) was noticed in 53.33% of patients in the dHACM allograft enhanced group on comparison with 33% in the controls. The overall results show that the augmentation of dHACM allograft to ACL reconstruction yields in good patient outcomes at post-operative follow-up.

## Introduction

Mobility requires a healthy knee with good function. The ACL is one of two ligaments that cross in the middle of the knee. One of the most frequent orthopaedic traumas is ACL damage, particularly in the population of professional athletes^1,2^. ACL tears occur on average at a rate of 68.6 per 100,000 person-years, with surgical reconstruction needed in the majority of cases^3^. ACL impairments in the knee can cause significant morbidity and long-term disability, which can lead to osteoarthritis, meniscal injuries, and chondral injuries^4,5^. Arthroscopic ACL reconstruction, the conventional gold standard treatment for ACL injuries, has developed over time with the aim of attaining a further anatomical and minimally intrusive repair procedure desiring to preserve stability and function with favourable outcomes^6^. For ACL reconstruction, either autograft or allograft may be employed^7^. Hamstring, Quadriceps, Peroneus longus tendons or a Bone patella tendon-bone (BPTB) autografts are frequently used Autografts. Graft harvest morbidity, biological incorporation failures, post-traumatic arthritis, and recurring injury, continue to be significant clinical issues^8^. Peroneus longus tendon autograft can be an appropriate autograft for ACLR due to its strength, larger graft diameter, and avoiding potential complications of hamstring autograft obtained from the knee region^9^. Rathomy *et al* reported that the peroneus longus tendon autograft harvesting had little effect on foot and ankle function^10^.

Many ACL rupture patients main treatment objective is to assist them quickly to return to their prior level of sport participation by minimising knee instability^11^. Even with an accelerated ACL rehabilitation program, the average time to return to sports is six to eight months^12,13^. Breakthroughs in tissue engineering have produced biologically enhanced ACL reconstruction methods using growth factors, stem cells, and scaffolds with the goal of enhancing biological integration and rejuvenation, minimising potential long-term side effects associated with ACL reconstruction, and maintaining the graft failure as low as possible^14^. Despite the paucity of level 1 trials, plate rich plasma (PRP) and bone marrow aspirate concentrate (BMAC) augmentations have been shown to be helpful for patients^15,16^. In recent times, hybrid grafts comprising of both autograft and allograft tissues were being widely employed owing to the benefits such as reduced risk of graft rupture and donor site morbidities after surgery^17^. Especially, the grafts typically prepared by using autologous peroneus tendon and soft tissue allograft, have attracted the attention of orthopaedicians for their usage in ACL reconstruction^18^. In this regard, this work describes the use of dehydrated Human Amnion/Chorion membrane (dHACM) augmentation in ACL reconstruction. dHACM naturally contains biological elements comparable to the PRP and BMAC. Prior research recognised over several growth factors, cytokine, chemokine, and protease inhibitors, majority of which functions work in stimulation of paracrine reactions in fibroblast, endothelial, and stem cells thereby promoting the tissue restorative processes^19^. Plantar fasciitis, ACL reconstruction, rotator cuff injury, tennis elbow, and Achilles tendinopathy have all recently been treated clinically with dHACM for tendons and ligament healing^19,20^. The dehydration of amnion and chorion membrane process removes moisture where enzymatic activity is reduced thus inhibiting the viability of microorganisms.

In order to achieve early and rapid recovery, we apply innovative tissue technology to improve intraosseous and intra-articular restorative process in ACL grafts used in reconstruction. The growth of Sharpey's fibres at the tunnel apertures assessed by MRI can be used to measure the augmentation. This case-control study examines the results of ACL tears that were surgically repaired with arthroscopic assistance employing a tripled peroneus longus autograft augmented with dHACM allograft membrane. The knee joints magnetic resonance imaging (MRI) is crucial for demonstrating how the transplanted ligamentous tissue changes during the healing process^21^. Post-operative MRI images were used to assess the ligamentization in the presence of augmented dHACM allograft. Generally, on a variety of animal models, the mechanical quality tests of grafts in ligamentization was assessed. These tests, however, do not apply in vivo. Quantifying the changes in the MRI signals and applying it in the clinical studies is the best noninvasive in-vivo technique. The strength of the MRI signals reflects the grafts histological remodelling. Signal-to-noise ratio, which measures the strength and spread of the signals in the grafts and tunnels, is frequently employed. In this study the ligamentization was determined using data from the MRI examination of the grafts, signal-to-noise ratio, clinical outcome scores, and clinical testing^21^. Overall favourable results in this controlled research with the augmentation of dHACM in ACL reconstruction suggest the potential for clinical translation.

## Materials and Methods

All of the patients (n=30) agreed to take part in the trial, provide clinical information, and have post-operative MRIs performed. This prospective randomised case control research, which received the approval of the local ethics committee in a private Multispeciality hospital in Pune. A total of 30 patients of both sexes with an isolated ACL tear were enrolled in the trial, and they undertook primary ACL reconstruction between June 2021 and September 2021. They also underwent a 6-month post-operative follow-up. The patients were divided into two groups. Group 1 (G1) consisted of 15 patients treated by ACL graft augmented with dHACM allograft, and Group 2 (G2) consisted of 15 patients treated by normal ACL reconstruction. The inclusion criteria were (i) age (18–50 years), (ii) ACL injury with/without meniscal tears, (iii) injury period (≤3 months), and (v) complete range of motion prior to surgery. The exclusion criteria were (i) any prior knee surgery or open knee injury, (ii) joint infections, (iii) the presence of skin lesions in or around the knee joints, such as psoriasis or eczema, (iv) metabolic bone diseases, (v) the use of drugs that affect bone turnover, such as Phenytoin, (v) patients who were not keen to sign the research consent form, and (vi) surgery within three weeks of injury. In this investigation, the patients were split into two groups at random.

The patient's ACL tear is confirmed based on the patients’ medical history, medical examinations, and radiological imaging, including magnetic resonance imaging (MRI). A three Tesla MRI [Seimens Magnetom Vida machine] was employed in this study. Patients with ACL tears are normally sportspersons who suffer a non-contact pivoting injury during a sudden change in direction and quick acceleration or deceleration. The most common complaints are typically a painful, swelling knee and trouble bearing weight. The Lachman and anterior drawer tests performed during a physical examination typically reveal a malfunctioning knee. The tibial plateau bone avulsion fracture is occasionally coupled with normal results on radiographs of the knee, which are generally normal. An MRI scan is often used to confirm the diagnosis of an ACL rupture. This scan will display ligament disruption along with any probable related meniscus/osteochondral abnormalities.

A single surgeon used the same surgical approach for all ACL reconstructions as per standard operation protocol. The difference in two groups lies in graft preparation. ACL Peroneus graft harvest and preparation with dHACM augmentation was carried out as per grouping of the patients. Using a sealed tendons stripper, a 3cm cut was made longitudinally over the lateral malleolus to harvest the Peroneus longus tendon from the ipsilateral side. On the back table of the operation theatre, the right-sized tendon autograft harvests are processed. To assess the graft diameter and guarantee adequate fit, the graft is tripled on the length of the suture strand. The typical graft diameters were 9.0, 9.5, and 10mm. After that, the graft is looped through the adjustable Endobutton.

**Fig 1:**
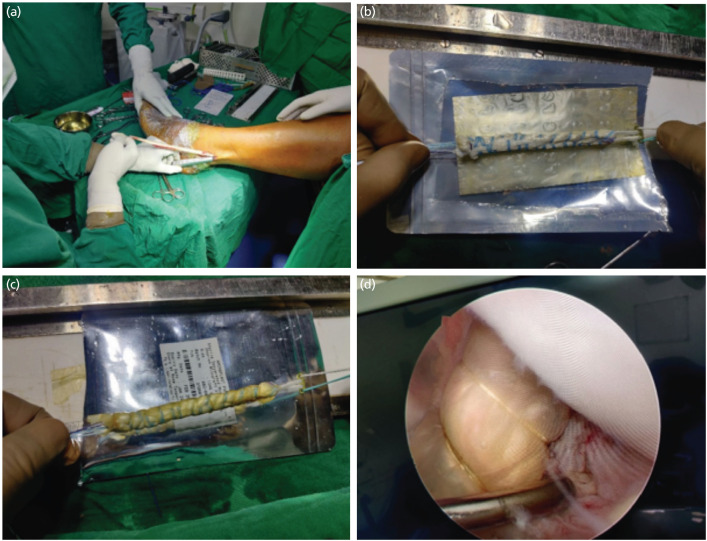
dHACM augmented ACL graft preparation. (a) Harvesting Peroneus Longus graft as per standard protocol. (b) Weaving the graft using fibrewire and wrapping of dehydrated Amnion chorion Membrane using monocryl sutures. (c) Final graft prepared after wrapping. (d) Intra-articular view of dHACM augmented ACL graft.

For patients in Group 1, an 8x6cm dHACM allograft was wrapped around the graft like a sleeve and is mildly rehydrated with sterile saline solution. To attach the membrane to the graft, a No. 2-0 Monocryl suture in wraparound mode was used to stitch the dHACM allograft into place. To enable superficial connection with the femoral notch and tibial tunnel, the dHACM allograft matrix extents the tendon graft from the proximal to distal end. The images of graft preparation are shown in Fig. 1.

Diagnostic arthroscopy is performed in all cases to confirm the ACL tear. To aid in the tunnel placement and femoral fixation, place a beath pin in the right place, the ACL femoral foot print area is debrided with a No. 4-0 shaver. By raising the leg, the knee is flexed past 90°, and an endoscopic 4.5mm drill pin is then shot through the femoral condyle. After measuring the depth of the bone, the pin is then advanced through the skin. An appropriate-depth tunnel is drilled using a femoral reamer of the proper size, parting at least 5 to 10mm of bone amid the graft and button. The knee is then flexed to 90°, and a 3cm incision is made 2.5cm distal to the medial joint line, halfway between the tibial tubercle and Gerdy's tubercle. The targeted guide is positioned and locked in the anteromedial portal at the tibial ACL footprint, and the tibial drill guide, set to 55°, is inserted via the tibial incision. The anterior horn of the lateral meniscus's posterior aspect is lined up with the tibial ACL footprint, and a drill pin is discharged at that location. The pin is then used as a guide to drill a hole through the tibial tube that is the same diameter as the graft. Using cautery, soft tissue is removed from the tibial tunnel anterior opening before being cleaned with sterile gauge. The pin and free ends of the suture are removed laterally, and the loop of the suture is then pushed via the tibial tunnel to the anterior surface of the tibia. The suture is free to pass through the eyelet of the beath pin, the antero-medial portal, and the femoral socket.

In Group 1 the adjustable endobutton with Peroneus longus graft along with dHACM allograft is advanced through the tibial and femoral tunnels where as, in Group 2 Peroneus longus graft alone with adjustable endobutton in using the passage suture that has been pulled through the tibial tunnel.

To conduct the clinical inspection and post-operative care and evaluate the knee stability, tests like the Lachman and pivot-shift are used. To confirm that the ACL button rests flush on the lateral femoral cortex and that the required tibial tunnel trajectory is present, a final intra-operative fluoroscopic image of the distal femur and proximal tibia is acquired. The extra ACL graft is then removed, leaving the tibial tunnel cortex flush. The wounds are irrigated as usual and then stitched up. After surgery, the knee is immediately locked in extension and kept there until the regional anaesthesia wears off.

Later, the knee was only kept extended while sleeping and using a walker to support complete weight bearing. By the 14th day following surgery, when the sutures were removed, the patients were urged to gradually increase their range of motion up to 90°. Patients were permitted to walk unassisted at one month but with a knee brace until three months after surgery. Jogging was started at four months after surgery, and sports-specific drills were permitted at six months after surgery. At eight months following surgery, full participation in sports was permitted, hence 8th month follow-up is deemed for the final clinical result. Both groups underwent MRI scans at six months after surgery to check for ligamentization of the graft, tunnel widening, and tunnel position, as well as to look for any complications as an intermediate study follow-up.

To evaluate the outcomes of ACL reconstruction surgery, the Tegnor-Lysholm score scale was employed. The scale included the following eight parameters: (i) limp; (ii) support; (iii) locking; (iv) instability; (v) pain; (vi) swelling; (vii) stair climbing; and (viii) squatting^22^.

## Results

**Fig 2:**
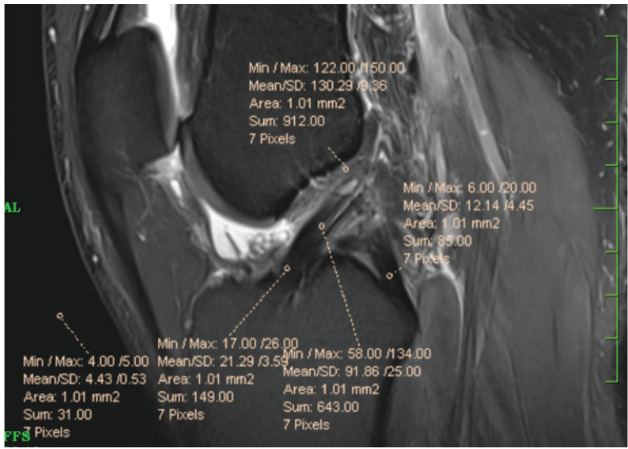
Calculation of average signal for ACL Ligamentization by taking average of numerator in mean by/SD ratio. For example, here mean signal value is 130.29+91.86+21.29/3 = 243.44/3 = 81.14

No substantial differences were noticed among the two groups in terms of age, sex, graft width, graft length, tunnel diameters, fixation devices, and post-operative regimen (Table I). Statistical T test were used to calculate p-value. The average age of the participants in this work was 30.3±7.5 years. The average time from damage to surgery was 1.1±0.3 months. Graft length and graft width on average were 125.9±9.1mm and 9.8±0.7mm, respectively. The average period for graft curing in the control group was 8.6 months, whereas it took 5 months in the groups that had dHACM allografts added. The MRI results were used to calculate the signal-to-noise-ratio. The signal was taken as nominator of the Mean/SD in ROI circle of 1mm^2^ area were calculated over MRI gantry over origin, mid substance and insertion points of ACL as shown in the Fig. 2. The air in front of Tibia and PCL insertion points were used as reference. The signal average numbers are considered as follows: (i) ≤75 – good healing; (ii) 75 to 150 – moderate healing; (iii) ≥150 – poor healing. In six-month post-operative MRI, in the G1 group, nine patients showed good healing, four patients showed moderate healing, and two patients showed poor healing. However, in the G2 group, good healing was seen in five patients, moderate healing in six patients, and poor healing was witnessed in four patients. When compared to the control group (G2), which did not receive any augmentation (33.33%), the majority of patients in the dHACM allograft enhanced group (G1) had ligamentization (SNR value <75) at six months MRI (53.33%) (Table II). SNR ratio value is significantly (p = .047) higher in G2 than in G1 The mean Lysholm Knee scores were assessed two, four, six, and eight months after surgery (Table III).

**Table I:** Patient demographics.

Patients	Age	Sex	Side	Post injury status	Pre-operative	Lysholm scores			
						2nd Month	4th Month	6th Month	8th Month
Study group (G1)					46.3	67.6	70.6	87.3	92.3
P1	26	M	Right	30 days	40	69	72	88	93
P2	32	M	Left	28 days	52	61	62	79	86
P3	46	M	Right	60 days	48	72	78	85	95
P4	30	F	Right	30 days	43	50	52	86	94
P5	28	M	Left	45 days	46	62	60	82	86
P6	23	M	Left	21 days	62	75	83	96	96
P7	33	F	Right	26 days	26	48	57	82	88
P8	41	F	Right	35 days	33	68	68	83	86
P9	37	M	Left	40 days	50	79	82	92	98
P10	31	M	Right	50 days	28	64	67	89	96
P11	47	M	Left	28 days	49	84	82	90	92
P12	24	M	Right	26 days	63	73	77	84	88
P13	20	F	Left	45 days	20	59	63	88	91
P14	29	F	Left	28 days	72	80	84	90	96
P15	24	M	Right	30 days	63	69	70	94	100
Control group (G2)					45.0	69.3	68.6	79.66	83.3
P16	30	M	Left	35 days	31	70	68	74	78
P17	28	M	Right	21 days	39	79	82	86	88
P18	36	F	Left	26 days	65	73	77	80	82
P19	42	F	Left	60 days	26	62	63	82	84
P20	24	M	Right	45 days	71	80	54	83	86
P21	27	M	Right	50 days	62	69	72	84	91
P22	43	M	Left	34 days	47	79	82	88	90
P23	48	M	Left	28 days	29	64	67	89	92
P24	35	F	Right	55 days	40	71	52	76	78
P25	25	M	Right	20 days	43	61	63	72	76
P26	38	F	Left	25 days	49	72	74	81	85
P27	26	M	Left	40 days	41	62	68	73	75
P28	23	M	Left	35 days	43	68	72	81	86
P29	22	M	Right	52 days	62	80	83	84	89
P30	27	F	Right	22 days	27	50	52	62	70

**Table II:** MRI evaluation of ACL based on SNR value.

	Study group (G1)	Control group (G2)
Good Healing (Signal Average <75)	9 subjects	5 subjects
Moderate Healing (Signal Average 75 to 150)	4 subjects	6 subjects
Poor Healing (Signal Average >75)	2 subjects	4 subjects

**Table III:** Average evaluation scores of patient groups throughout the study.

Variables		Groups	N	Mean	Std. Deviation	P value
Age		Case	15	31.4000	8.20105	0.947
		Control	15	31.6000	8.17487	
SNR_ratio_signalvalue		Case	15	60 79	92.63232	0.047
		Control	15	104.86	56.76445	
Lysholm Score	Second month	Case	15	67.5333	10.40513	0.608
		control	15	69.3333	8.53285	
	Fourth Month	Case	15	70.4667	10.30164	0.626
		Control	15	68.6000	10.41839	
	Sixth Month	Case	15	87.2000	4.79881	0.002
		Control	15	79.6667	7.14809	
	Eighth Month	Case	15	92.3333	4.63938	<.0001
		Control	15	83.3333	6.59726	

The average pre-operative Lysholm score was 46.3 and 45.0 in dHACM augmented (G1) and standard (G2) groups, respectively. At two months and four months, there is no apparent difference among the two groups. Whereas the post-operative scores at six-month follow-up were 87.2 and 79.6 (p-value=0.002), and eight-month follow-up scores were 92.3 and 82.3 (p-value <0.0001), respectively, in G1 and G2 groups which is significant.

## Discussion

Although common, a complete rupture of the ACL can be a serious setback for any patient, but athletes in particular may suffer because of it. The accompanying treatments have undergone much research over time and have developed into the variety of alternatives available to surgeons today. In addition to deciding whether to operate after an ACL tear, the treating doctor must also decide what kind of graft to utilise and when the athlete should resume sporting activities. Even if it varies on a number of factors, the latter is determined by the level of post-operative graft maturation through the complex process of recollagenization and rise in mineralisation after the initial inflammatory reaction typical of wound healing^23^.

The most important results of this study indicate that tripled Peroneus autograft augmented with dHACM allograft-assisted ACL restoration had a decent clinical outcome with a significant difference between the two groups. Tendon-to-bone healing of an ACL that has undergone reconstruction is crucial for lowering the chance of failure in a patient. ACL reconstruction failure can have a variety of causes, but there are three primary medical signs and indications including unsteadiness, persistent effusion, and chronic pain that indicate failure. By enhancing biological integration and lowering the post-operative inflammatory reaction, amnion augmentation lessens these indications^24,25^. In wound care, ophthalmic and plastic surgeries, amniotic membrane-derived products have been examined and proved to be effective, however, amnion-resultant products have not seen much use in ligament replacement^26^.

It has been reported that the dHACM contains biological elements related to platelet-rich plasma and bone marrow aspirate concentrate. The dHACM production is patented (PURION) and the process comprises mild cleaning of human amnion and chorion membrane followed by lamination and dehydration of the tissues in precise settings. Both the membrane form and the micronised form of 25 dHACM allografts can be applied, the latter is made of membrane tissues that were subjected to cryomilling and sieving using 180 and 25mm sieves for particle size. Usually, a saline solution is used to reconstitute the micronised tissue before giving it to patients as a flowable allograft. Over 226 growth factors, cytokine, chemokine, and protease inhibitors were discovered in earlier investigations. Several of these agents activate paracrine reactions in fibroblast, endothelial, and stem cells to aid in tissue healing and restoration. For the treatment of plantar fasciitis, ACL renewal, rotator cuff injuries, tennis elbow, and Achilles tendinopathy, clinical uses of dHACM for tendon and ligament tissue healing were lately employed. The removal of moisture from the amnion and chorion membrane process reduces enzymatic activity and impairs microbial viability^27^.

The amniotic membrane constantly changes as the embryo grows, giving it the natural capacity to hold onto a reserve of stem cells all over the prenatal period. Compared to stem cells obtained from embryonic tissue, amniotic derivative stem cells have the particular benefit of not undergoing teratogenic transformation or endangering the foetus. The amniotic membrane produces two different types of stem cells: amniotic epithelial cells (AEC) and amniotic mesenchymal stromal cells (AMSC). While AMSCs arise from mesoderm and can differentiate into mature lineages, AECs can distinguish into three germ layers (mesoderm, endoderm, and ectoderm). While AMSCs arise from mesoderm and can differentiate into mature lineages, AECs can discern into all three germ layers (mesoderm, endoderm, and ectoderm). For orthopaedic tissue engineering, AECs and AMSCs can both develop into mature cell lineages^28,29^.

It has been demonstrated that the amniotic membrane possesses anti-inflammatory effects. The absence of chief histocompatibility antigens and the generation of anti-inflammatory cytokines are two factors that contribute to the amniotic membrane's intrinsic immunomodulatory capabilities. Both the inherent and adaptive immune systems are impacted by the amniotic membrane stem cells. Reduced antigen production, resistance to natural killer cell lysis of stem cells, decreased T cell proliferation, and varying impacts on the level of circulating cytokines are all results of these effects. Additionally, the amniotic membrane's anti-inflammatory qualities have been linked to its capacity to actively reduce adhesion formation and support scar less recovery. Transforming growth factor-b, which is known to cause fibrotic reactions by activating fibroblasts, is blocked by the amniotic membrane. These features of the amniotic membrane taken as a whole have been suggested to have a multifaceted impact on wound healing^30^.

The use of amnion in orthopaedic problems has showed encouraging results in a number of in vitro experiments and in vivo animal investigations. Amnion tendon covering uses have also been documented, with research demonstrating lower impediment rates, lower pain levels, and higher functional results. Further research should be done to ascertain whether the use of amnion biological augmentation can enhance healing in ACL restoration through anti-inflammatory, scaffolding, and stem cell-producing actions^26^.

Limitations of study is this was conducted in a small cohort of 30 patients with 15 controls and 15 cases with age matched. Ideally 30 controls should be there for 15 cases maintaining a ratio of 2:1 in case of case control study as per STROE MR guidelines. The MRI signal to Noise ratio method based on signal to noise ratio has not be standardised. Very few studies document the ligamentation with the help of Noise to signal ratio^21^. Multicentric largers studies would open new avenues of research. This case control series would be small step towards such multicentric trails with larger numbers.

## Conclusion

Tendon-to-bone healing of a reconstructed ACL is of tremendous importance after ACL surgery to decrease a patient’s risk of ACL reconstruction failure. By comparing the outcomes of the two groups in our study population, we were able to draw the conclusion that dHACM allograft demonstrated quicker and better graft healing in ACL restoration. Thus, the clinical and radiological study confirms the best use of biologic such as dHACM in graft healing to help in the early return to sports and is recommended for athletes.
